# Bilateral Axillary Lymphadenopathy After COVID-19 Vaccine Presenting for Lymph Node Surgical Biopsy: A Case Report

**DOI:** 10.7759/cureus.35834

**Published:** 2023-03-06

**Authors:** Amanda Taylor, John Michael, John Sciarra, Andrzej Kuchciak, Mohammed M Masri

**Affiliations:** 1 Anesthesiology, Larkin Community Hospital, South Miami, USA; 2 School of Medicine, Lake Erie College of Osteopathic Medicine (LECOM), Tampa, USA; 3 General Surgery, Larkin Community Hospital, South Miami, USA

**Keywords:** covid-19, coronavirus, vaccination reaction, covid, coronavirus pandemic, axillary lymphadenopathy, covid-19 mrna vaccine, pfizer-biontech covid-19 vaccine, covid-19 vaccine, bilateral axillary lymphadenopathy

## Abstract

The coronavirus disease 2019 (COVID-19) pandemic ravaged China, made its way to Thailand and Japan, and ultimately spread across the globe. Despite all efforts to contain the virus, hundreds of millions of positive cases and millions of deaths have been reported worldwide. Due to the vastness and severity of this virus, there was a desperate need for a vaccine, quickly. The COVID-19 vaccination was created urgently under emergency use authorization (EUA) by the US Food and Drug Administration (FDA) in less than one year, a process typically taking over 10 years. With this expedited creation time, there is also a shortened time frame for clinical trials, which is commonly used to evaluate for effectiveness and identify any potential side effects or adverse reactions to the created vaccine. We will discuss some potential side effects of receiving the Pfizer-BioNTech COVID-19 mRNA vaccination. In this case report, we discuss one individual who received two doses of the Pfizer-BioNTech COVID-19 mRNA vaccine and experienced a previous unreported adverse side effect of non-self-remitting bilateral axillary lymphadenopathy. This reaction was not originally seen during the clinical trial phase of the vaccine creation, which caused this individual to obtain a full medical workup including ultrasound, computed tomography (CT) scans, and blood work and ultimately needing surgical intervention to have the axillary lymphadenopathy excised. We aim to shed light on a new, undocumented adverse reaction that should be included in physicians’ differential diagnoses in individuals after receiving the COVID-19 vaccine, particularly the Pfizer-BioNTech COVID-19 mRNA vaccination. This information could help future patients avoid unnecessary extensive medical workups, surgical procedures, being exposed to anesthesia, or having the burden of additional unwarranted healthcare costs.

## Introduction

The first human case of coronavirus disease was identified in late 2019 and is speculated to have originated in Wuhan, China. At that time, no one anticipated how rapidly this virus would spread. In less than three months, the virus had ravaged China and made its way to parts of Thailand and Japan, ultimately spreading across the globe despite all efforts to contain the virus. In a matter of months, the coronavirus disease had turned into a global pandemic, referred to as COVID-19 [1]. Now, almost 24 months after the first positive human case, the medical community is still immersed in the COVID-19 pandemic with over 217 million confirmed positive cases and over 4.5 million deaths reported worldwide [2].

This prompted the first-ever cause for emergency use authorization (EUA) by the US Food and Drug Administration (FDA) for the creation of vaccination against COVID-19 [3]. Due to the severity of the global pandemic and with this emergency use authorization issuance, an entirely new, never-before-used vaccine type was developed in record time. Once the genetic code of the virus was released in January 2020, several manufacturing pharmaceutical companies were underway to create a vaccine. Within six weeks, a vaccine was generated and ready to undergo clinical trials. Within six months, all three phases of clinical trials were completed, and the US Food and Drug Administration approval was requested [4].

In a matter of six months, a new approach to vaccines using mRNA was created. Put simply, mRNA vaccines teach our cells how to make a specific protein that triggers an immune response. This immune response produces antibodies that protect us from getting infected if we encounter the actual virus [5]. This new approach varies in the fact that usually a weakened or inactivated virus particle is injected to trigger an immune response as opposed to injecting mRNA.

A process that typically takes over 10 years was completed in less than one year. According to the Food and Drug Administration, although the production of a vaccine was expedited under a government emergency, the approved vaccines have been held to the same rigorous safety and effectiveness standards as all other types of vaccines in the United States. The only COVID-19 vaccines the FDA will make available for use in the United States are those that meet these standards [4]. Here, we will focus on one FDA-approved mRNA vaccine: the Pfizer-BioNTech COVID-19 mRNA vaccine. The Pfizer-BioNtech COVID-19 vaccine is an intramuscular two-dose vaccine given 21 days apart, targeted for individuals 12 and older. Since the approval of the vaccines, there have been over five trillion vaccine doses administered to the public worldwide.

Due to the expedited production and validation time, it has been challenging for physicians and patients alike because of newly emerging adverse effects. The most commonly reported side effects after administering the vaccine include pain at the injection site, fatigue, headache, fever, chills, muscle pain, and/or joint pain [2,4,6]. Reports of lymphadenopathy in the Pfizer-BioNTech COVID-19 group revealed 58 more cases in the vaccine group (64) than in the placebo group [6]. Consequently, lymphadenopathy is plausibly related to the vaccine. Lymphadenopathy occurred in the arm and neck regions and was reported within two to four days after vaccination. The average duration of lymphadenopathy was approximately 10 days [7].

It is important to note that hyperplastic axillary lymph nodes can be seen on sonography after any vaccination but are more common after a vaccine that evokes a strong immune response, such as the COVID-19 vaccine [2,3,8]. Unilateral axillary lymphadenopathy on the ipsilateral side of the injection site was a very common side effect [2,4,9]. Although many adverse side effects have been reported, after extensive research, it appears that bilateral lymphadenopathy is extremely uncommon, and subsequent data is missing from the literature.

In this case report, the objective is to potentially present the first case of bilateral axillary lymphadenopathy following the second dose of the Pfizer-BioNTech COVID-19 vaccine. In this case report, a Hispanic male (registered nurse) developed bilateral axillary lymphadenopathy days after receiving the second dose of the COVID-19 vaccine with enlargement over three to four months and no regression seven months after the second dose of the vaccine was administered.

## Case presentation

A 62-year-old Hispanic male, with a past medical history of well-controlled hypertension and gastroesophageal reflux, who is a registered nurse (referred to as the patient), received his first dose of Pfizer-BioNTech COVID-19 vaccination on December 22, 2020. At this time, the FDA issued the first emergency use authorization (EUA) for the use of the Pfizer-BioNTech COVID-19 vaccine. This vaccine was recommended for people aged 16 years and older. Healthcare personnel and long-term care facility residents were offered COVID-19 vaccination first (phase 1a) [10].

**Figure 1:**
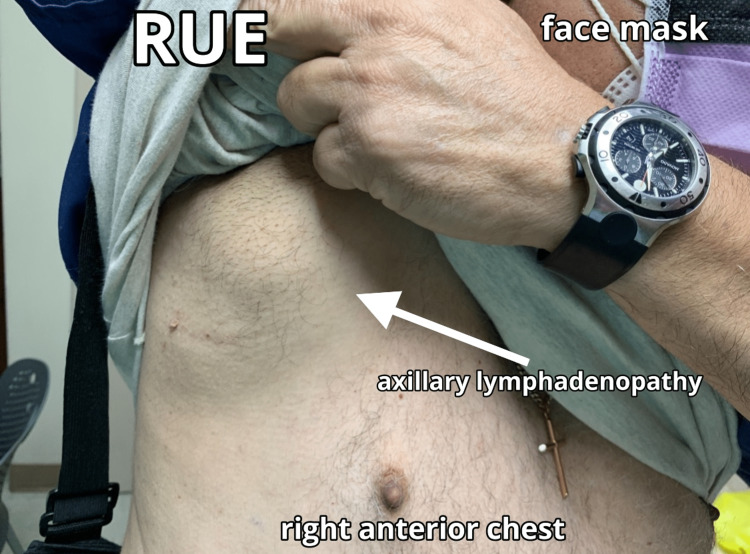
Initial presentation of right axillary swelling noted on 1/14/21, two days after the second dose of the Pfizer-BioNTech COVID-19 vaccine COVID-19: coronavirus disease 2019, RUE: right upper extremity

Immediately following the vaccination administration intramuscularly in his right upper extremity, the patient experienced pain at the injection site with no additional adverse side effects. Twenty-one days later, on January 12, 2021, the patient was inoculated again in his right upper extremity with the second dose of the Pfizer-BioNTech COVID-19 vaccine. After receiving the second dose, the patient started to experience his unique side effects. Beginning on day 0, the day the second dose of the vaccine was administered, the patient noted slight pain at the injection site. This localized pain was less when compared to the first injection received 21 days prior. On day 1 after the second vaccine, the patient felt subjective fever and chills and took 800 mg of acetaminophen every six hours for a total of two doses and noted improvement. On day 2, the patient had self-identified painless lymphadenopathy under his right axilla (Figure 1) and was initially noted to be small and mobile, with an estimated size of 1 cm × 1 cm. Over the next 30 days, the patient noticed axillary lymphadenopathy starting to develop in his left axilla as well, contralateral to the side of the injection. Over the next three to four months, bilateral axillary masses gradually grew larger with the left becoming slightly tender to palpation and increasing in firmness. It is also important to note that the patient has never experienced side effects like this in the past following vaccinations. Since the patient is a healthcare worker, he annually receives influenza vaccination with no similar side effects. The patient also received the Bacillus Calmette-Guérin (BCG) vaccination when he was a child, again with no similar side effects of lymphadenopathy.

Four months after his second vaccination, when it was apparent that the patient’s bilateral axillary lymphadenopathy was not self-limiting, he decided to seek medical attention from his primary care physician. It was recommended that the patient obtain a non-contrast chest computed tomography (CT) scan of the left axilla along with routine blood work. The imaging study was performed, and blood samples were collected on May 24, 2021, four months after the administration of the second dose of the COVID-19 vaccination.

The CT scan identified no axillary lymphadenopathy. However, soft tissue swelling was observed with calcified encapsulation. Initially, there was a concern for malignancy such as lymphoma or leukemia, multiple myeloma, any other systemic disease, or infection. Complete blood count (CBC) with differential was within normal limits, including a white blood count of 8.7 × 10^9^/L, hemoglobin of 16.2 g/dL, hematocrit of 46.9%, and platelets of 147 × 10^9^/L. Serum immunofixation (IFE) and electrophoresis (EP) revealed that all immunoglobulins were within normal limits as well. The cholesterol panel was grossly unremarkable with cholesterol at 173 mg/dL, triglycerides elevated at 272 mg/dL, high-density lipoprotein (HDL) at 40 mg/dL, and low-density lipoprotein (LDL) at 88 mg/dL. The results of the SARS-COV-2 antibodies identified a level of 929 u/mL, suggesting adequate antibody creation to the virus after the administration of the vaccine; it is considered a negative interpretation if the value is <0.8 u/mL (Table 1).

**Table 1:** Laboratory results for the patient’s blood sample obtained after identifying bilateral axillary lymphadenopathy WBC: white blood count, Hgb: hemoglobin, Hct: hematocrit, HDL: high-density lipoprotein, LDL: low-density lipoprotein, SARS-CoV-2: severe acute respiratory syndrome coronavirus 2, L: liter, g/dL: grams per deciliter, %: percentage, mg/dL: milligrams per deciliter, u/mL: units per milliliter

Laboratory results	Results	Reference interval	Units
WBC	8.7	4.5-10.5	10^9^/L
Hgb	16.2	13-18	g/dL
Hct	46.9	41-50	%
Platelets	147	150-400	10^9^/L
Cholesterol	173	Low, <200	mg/dL
Triglycerides	272	High, 200-499	mg/dL
HDL	40	Desirable, >40	mg/dL
LDL	88	Optimal, <100	mg/dL
SARS-CoV-2 antibodies	929	Negative result, <0.8	u/mL

**Figure 2:**
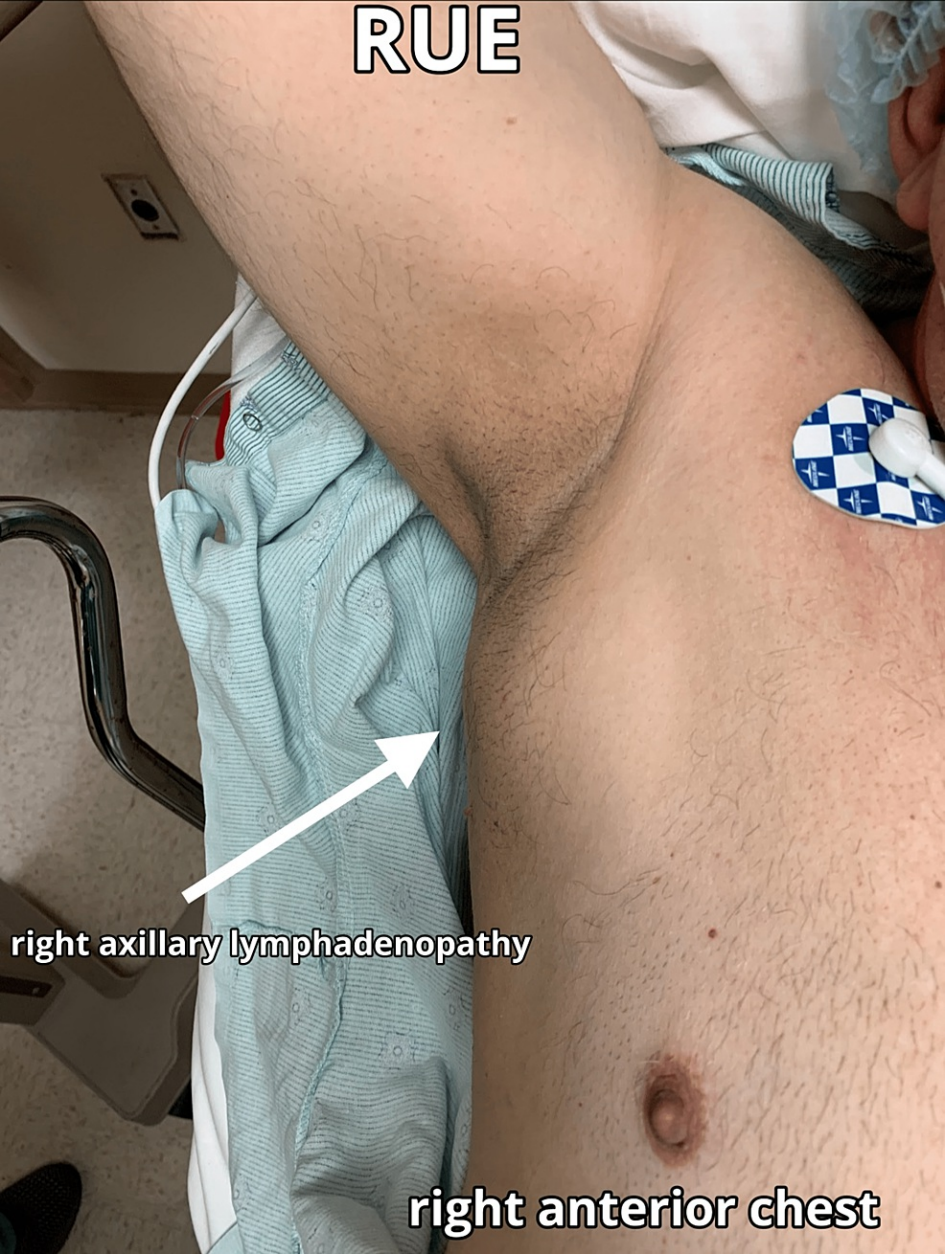
Final presentation of right axillary swelling on 8/26/21, seven months after the second dose of the Pfizer-BioNTech COVID-19 vaccine The RUE is extended laterally, raised near the patient’s head with the arrow pointing to the area of swelling. COVID-19: coronavirus disease 2019, RUE: right upper extremity

**Figure 3:**
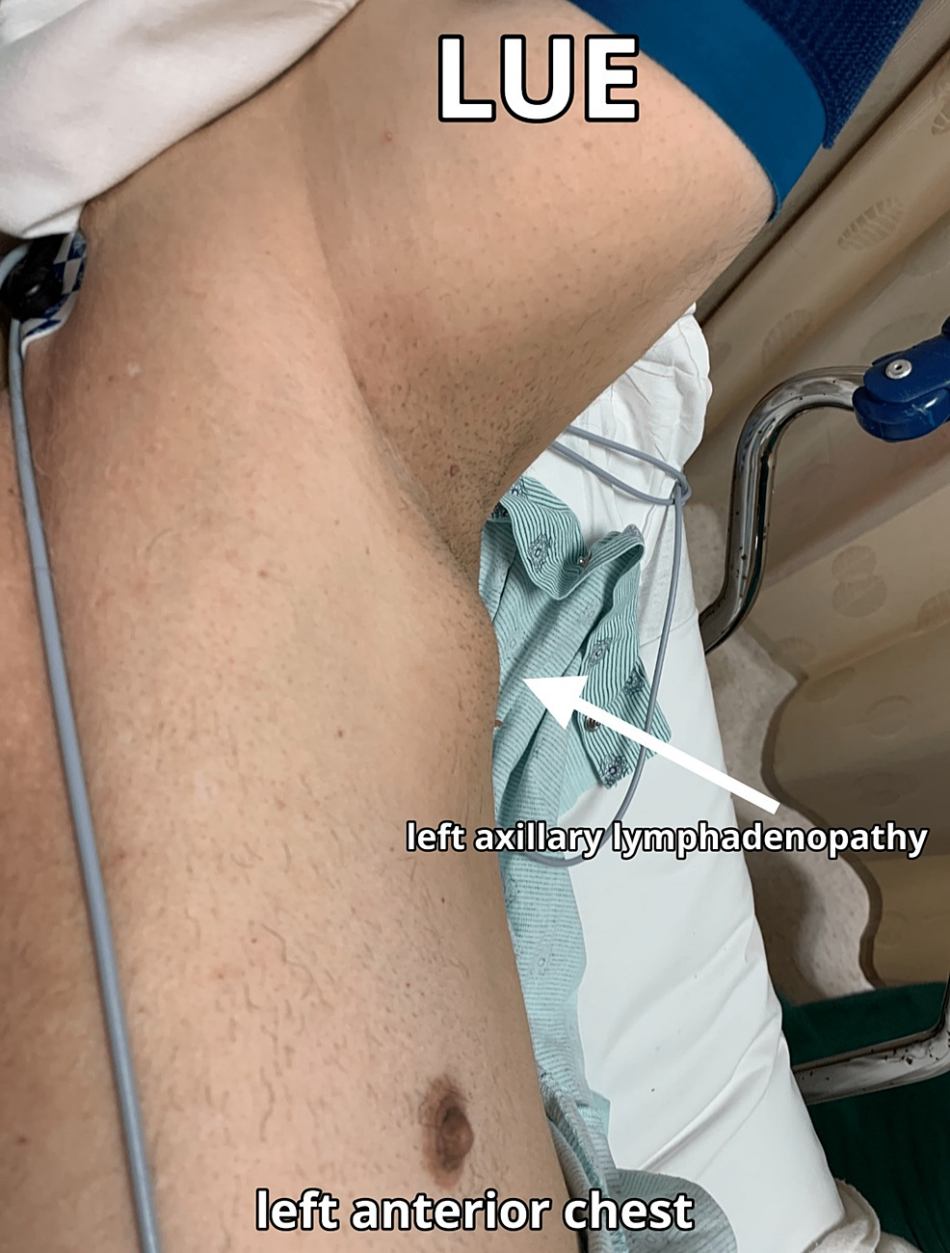
Final presentation of the LUE axillary swelling on 8/26/21, seven months after the second dose of the Pfizer-BioNTech COVID-19 vaccine The left upper extremity is extended laterally with a blue blood pressure cuff around the patient’s bicep and raised near his head with the arrow pointing to the area of swelling and the surgical site. COVID-19: coronavirus disease 2019, LUE: left upper extremity

Now, seven months after the second dose of the Pfizer-BioNTech COVID-19 vaccine, the bilateral axillary lymphadenopathy continued to remain stable in size with no regression noted and no significant changes identified. The bilateral axillary lymphadenopathy was measured, and it was estimated that the right axillary mass externally measured 9 cm × 5 cm (Figure 2), and the left axillary mass externally measured 7 cm × 6 cm (Figure 3). The patient reported no additional symptoms except the ones previously mentioned.

With the seemingly normal laboratory findings and imaging, the patient’s primary care physician was starting to narrow their differential. It still could not be fully ruled out that this was a potential malignancy due to the uncharacteristic nature of the persistent, non-self-limiting, bilateral axillary lymphadenopathy. It was then that the patient sought out further medical attention, in the form of a general surgeon. The general surgeon recommended surgical resection of the left axillary mass with the specimen being sent to pathology as a frozen section to verify and rule out malignancy.

Upon arrival at the operating room, a complete history was obtained. The procedure was performed under general anesthesia. For induction, fentanyl 100 mcg, propofol 150 mg, and rocuronium 50 mg were given. The patient was easy to mask ventilate and was intubated with direct laryngoscopy using a Mac-3 blade and insertion of a size 7.5 endotracheal tube; a grade 1 view was observed during intubation.

Throughout the entirety of the case, the patient was maintained on volatile anesthetics, a combination of sevoflurane (2%-3%) and nitrous oxide (50%) with oxygen (50%) at a flow rate varying from 2 to 3 L. The patient remained extremely hemodynamically stable throughout the case with a systolic blood pressure (SBP) ranging from 108 to 136 mmHg, a diastolic blood pressure (DBP) ranging from 68 to 82 mmHg, and a heart rate (HR) ranging from 72 to 89 beats per minute.

**Figure 4:**
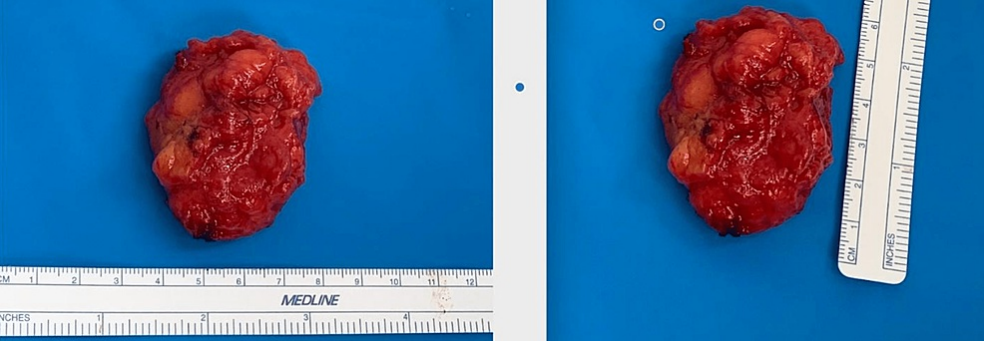
Two views of the solitary excised mass from the left axillary region measuring 4 cm × 6 cm

The patient tolerated the procedure well, and immediately following the removal of the right axillary lymph node (Figure 4), it was sent to pathology. The preliminary report while in the operating room revealed a fatty lymph node negative for malignancy.

Once the incision closure was completed, the patient was fully reversed with neostigmine 4 mg and glycopyrrolate 0.8 mg. The patient was ready for extubation and noted to have adequate minute ventilation, sustained head lift, and sustained tetanus. The patient was extubated without any complications and subsequently taken to the post-anesthesia care unit (PACU) where he was then observed for the next 60 minutes and was discharged the same day.

The patient had a follow-up appointment with the general surgeon one week later, where his surgical incision was healing well and the sutures were removed. The official pathology report was also available at this time and identified the mass as one benign lymph node with benign lipomatosis (Table 2).

**Table 2:** Frozen section: surgical pathology report of the left axillary mass that was surgically excised

Surgical pathology report	
Clinical history	Left axillary mass, mass excision with frozen section
Diagnosis	Left axillary mass, one benign lymph node with benign lipomatosis
Gross description	Received fresh is a 5 cm × 3 cm × 3 cm yellow-tan soft tissue mass with a smooth and glistening outer surface, and is inked in black; on serial section, it showed homogenous yellow-tan adipose tissue; a touch prep is prepared for intraoperative consultation; representative sections of the specimen are submitted in three pink cassettes
Intraoperative consultation	One fatty lymph node, negative for malignancy on representative touch prep, findings were relayed

At the one-week follow-up appointment, it was also noted that there was a left-sided fluid collection where the axillary mass was removed. A sterile needle was inserted, and the fluid collection was drained, removing a total of 50 cc of clear fluid that was determined to be a seroma. The patient was then seen weekly by the surgeon for close monitoring and further evaluation of the left axillary region. At the next four weekly visits, the seroma recurred and each time was drained with 50 cc removed. On the final weekly evaluation, the seroma was again drained, but only 10 cc of fluid was removed. Six weeks after the surgical procedure, there was no recurrence of the seroma. At his three-month follow-up appointment with the general surgeon and primary care physician, the patient did not have any recurrence of the left-sided axillary lymphadenopathy or seroma formation, and his surgical site is well-healed. The right axillary lymphadenopathy continues to remain present with no signs of regression.

## Discussion

COVID-19 vaccines have proven efficacious. The sheer number of recipients of the vaccine highlights the weight and magnitude of the situation. It is important that we highlight, monitor, diagnose, and differentiate benign side effects from dangerous medical conditions, especially due to the expedited nature of the vaccine creation and decreased duration of the clinical trials with the emergency use authorization. The most commonly reported side effects after the administration of the vaccine include pain at the injection site, fatigue, headache, fever, chills, and muscle and/or joint pain [2,5,6]. Reports of unilateral ipsilateral lymphadenopathy in the Pfizer-BioNTech COVID-19 vaccine group were also revealed at a rate of 0.3%, when compared to the placebo group, highlighting the uniqueness of bilateral lymphadenopathy, which was not observed during the clinical trials [7]. Consequently, lymphadenopathy is plausibly related to the vaccine with lymphadenopathy occurring in the arm and neck regions and was reported within two to four days after vaccination with an average duration of approximately 10 days [11].

As previously mentioned, hyperplastic axillary nodes can be seen on sonography after any vaccination but are more common after a vaccine that evokes a strong immune response, such as the COVID-19 vaccine [6]. Unilateral axillary lymphadenopathy on the ipsilateral side of the injection site was a common side effect [7]. The incidence of lymphadenopathy has been well-documented with previous vaccination efforts. In a large cohort of patients receiving the influenza vaccine, four out of 83 patients had unexpected fluorodeoxyglucose axillary node accumulations on imaging [4,9,8].

In clinical trials of the mRNA COVID-19 vaccination, lymphadenopathy ipsilateral to the site of injection had an incidence of 10.5% in Pfizer patients and 11.6% in Moderna patients, respectively. The higher incidence of lymphadenopathy among COVID-19 vaccine recipients compared to previous vaccines might indicate a superior immunogenic response. The mRNA vaccines developed to fight the pandemic exhibit their effect by using the hosts’ rRNA to transcribe COVID-19’s spike protein. Once the host produces and recognizes the foreign spike protein, the host begins to mount cells that respond to the pathogen. These immense immune reactions occur throughout the body, specifically, in lymphoid tissues, often causing them to swell and grow in size [12]. Self-remitting lymphadenopathy is a known side effect of vaccines and is often mistaken for malignancy.

However, given the temporal and spatial relationship of this case’s bilateral lymphadenopathy, further investigation was warranted. The differences in physical examination, ultrasound, and pathology observed in the lymph nodes, with the right axillary lymph node described as soft and pliable, with edema and fluid, while the left axillary lymph node as firm and tender, may provide insight into the mechanism of action in this given case.

All COVID-19 vaccinations are given intramuscularly. Given the proximity of the injection, we observed more traditional physical examination findings regarding lymph node characteristics on the right axilla compared to the left. Unilateral lymphadenopathy occurs almost exclusively ipsilateral to the injection sight; this prompted surgical resection of the contralateral, left axillary lymph node with pathological analysis to appreciate the novelty of this case [13].

Pathological examination revealed a benign neoplasm of fatty lymph node tissue and a lack of malignancy. While this specific case was harmless, physical findings such as these may be of concern to those with a history of malignancy and their providers. It should be noted that any lymphadenopathy was associated with higher serological antibody levels such as in this case. The patient’s SARS-CoV-2 semi-quantitative total antibody level was 929 u/mL (negative if <0.8 u/mL) [14-18].

After an exhaustive review of the medical literature, roughly hundreds of cases have been reported of unilateral axillary lymphadenopathy after receiving the COVID-19 vaccination, which is to be expected based on the data in the clinical trials [2,3]. However, symptomatic, non-self-limiting bilateral axillary lymphadenopathy after receiving the COVID-19 vaccine has yet to be documented in the scientific community. Although this case’s findings were benign, we hope that this case report will shed light on an additional side effect observed in patients who received the COVID-19 vaccination, specifically the Pfizer-BioNTech COVID-19. It appears that future physicians now have an additional differential diagnosis in regard to bilateral axillary lymphadenopathy with recent inoculation of the coronavirus vaccination [15-18]. The information in this case report could potentially help future patients avoid an unnecessary surgical procedure, being exposed to potential complications and anesthesia, or having the burden of additional unwarranted healthcare costs. This overall would cause less harm to the patient and less burden to the medical staff.

## Conclusions

The COVID-19 pandemic ravaged China, made its way to Thailand and Japan, and ultimately spread across the globe. Despite all efforts to contain the virus, it affected millions if not billions of people worldwide. With COVID-19 causing hundreds of millions of deaths, there was an urgent need for the development of a vaccine. Under the emergency use authorization by the US Food and Drug Administration, the Pfizer-BioNTech COVID-19 mRNA vaccine was created in less than one year, a process that typically takes over 10 years. With this expedited creation time, there is also a shortened time frame for clinical trials, which is commonly used to evaluate for effectiveness and identify any potential side effects or adverse reactions to the created vaccine. During the brief clinical trials, the most common reactions were identified to include pain at the injection site, fatigue, headache, fever, chills, muscle pain, and/or joint pain. When compared to the placebo group, reports of rare unilateral ipsilateral axillary lymphadenopathy were revealed. However, there was no mention of bilateral axillary lymphadenopathy, highlighting the uniqueness of this case report.

In this case report, we discussed one individual who received two doses of the Pfizer-BioNTech COVID-19 mRNA vaccine and experienced an unreported adverse side effect of non-self-remitting bilateral axillary lymphadenopathy. Due to the uniqueness of this adverse reaction, it prompted the clinicians and individual to obtain a full medical workup including ultrasound, computed tomography scans, and blood work, ultimately requiring surgical intervention to have the left axillary lymph node resected. We aim to arm physicians with an additional differential diagnosis for non-self-remitting bilateral axillary lymphadenopathy in individuals after receiving the Pfizer-BioNTech COVID-19 mRNA vaccination, particularly the Pfizer-BioNTech COVID-19 mRNA vaccine. This information could help future patients avoid unnecessary surgical procedures, being exposed to anesthesia, or having additional unwarranted healthcare costs.
